# Association of the Ratio of Early Mitral Inflow Velocity to the Global Diastolic Strain Rate with a Rapid Renal Function Decline in Atrial Fibrillation

**DOI:** 10.1371/journal.pone.0147446

**Published:** 2016-01-28

**Authors:** Szu-Chia Chen, Wen-Hsien Lee, Po-Chao Hsu, Chee-Siong Lee, Meng-Kuang Lee, Hsueh-Wei Yen, Tsung-Hsien Lin, Wen-Chol Voon, Wen-Ter Lai, Sheng-Hsiung Sheu, Ho-Ming Su

**Affiliations:** 1 Division of Nephrology, Department of Internal Medicine, Kaohsiung Medical University Hospital, Kaohsiung Medical University, Kaohsiung, Taiwan; 2 Division of Cardiology, Department of Internal Medicine, Kaohsiung Medical University Hospital, Kaohsiung Medical University, Kaohsiung, Taiwan; 3 Department of Internal Medicine, Kaohsiung Municipal Hsiao-Kang Hospital, Kaohsiung Medical University, Kaohsiung, Taiwan; 4 Faculty of Medicine, College of Medicine, Kaohsiung Medical University, Kaohsiung, Taiwan; 5 Graduate Institute of Clinical Medicine, College of Medicine, Kaohsiung Medical University, Kaohsiung, Taiwan; Temple University, UNITED STATES

## Abstract

The ratio of early mitral inflow velocity (E) to the global diastolic strain rate (E’sr) has been correlated with left ventricular filling pressure and predicts adverse cardiac outcomes in atrial fibrillation (AF). The relationship between the E/E’sr ratio and renal outcomes in AF has not been evaluated. This study examined the ability of the E/E’sr ratio in predicting progression to the renal endpoint, which is defined as a ≥ 25% decline in the estimated glomerular filtration rate in patients with AF. Comprehensive echocardiography was performed on 149 patients with persistent AF, and E’sr was assessed from three standard apical views using the index beat method. During a median follow-up period of 2.3 years, 63 patients (42.3%) were reaching the renal endpoint. Multivariate analysis showed that an increased E/E’sr ratio (per 10 cm) (hazard ratio, 1.230; 95% confidence interval, 1.088 to 1.391; *p* = 0.001) was associated with an increased renal endpoint. In a direct comparison, the E/E’sr ratio outperformed the ratio of E to early diastolic mitral annular velocity (E’) in predicting progression to the renal endpoint in both univariate and multivariate models (*p* ≤ 0.039). Moreover, adding the E/E’sr ratio to a clinical model and echocardiographic parameters provided an additional benefit in the prediction of progression to the renal endpoint (*p* = 0.006). The E/E’sr ratio is a useful parameter and is stronger than the E/E’ ratio in predicting the progression to the renal endpoint, and it may offer an additional prognostic benefit over conventional clinical and echocardiographic parameters in patients with AF.

## Introduction

Atrial fibrillation (AF) is the most common form of cardiac arrhythmia and its prevalence increases with age, reaching a prevalence rate of 8% in individuals older than 80 years [1, 2]. Furthermore, AF is associated with an increased risk of stroke, heart failure, and all-cause mortality [3]. Recently, we found that anemia and echocardiographic systolic and diastolic parameters from traditional echocardiograhy are useful predictors of cardiovascular outcomes in patients with AF [4]. In patients with AF, a relative reduction (≥ 25%) in the estimated glomerular filtration rate (eGFR) independently predicts the risk of stroke and death [5]. Therefore, identifying AF patients with rapid renal function progression for aggressive treatment interventions is crucial in disease attenuation and prolonged survival.

Echocardiographic measures of left ventricular (LV) function and structure have been reported to predict adverse renal outcomes in advanced chronic kidney disease (CKD) patients from 2-D and tissue Doppler echocardiograhy [6, 7]. We have reported that impaired LV systolic and diastolic functions were associated with a rapid renal function decline and progression to dialysis in patients without AF [7, 8]. The LV early global diastolic strain rate (E’sr) was reported to be a useful parameter of an LV diastolic function [9]. Furthermore, several studies have demonstrated a close correlation between invasively measured LV filling pressure and the ratio of early mitral inflow velocity (E) to E’sr [10–12]. Recently, we reported that the E/E’sr ratio was associated with adverse cardiac events in patients with AF [13]. However, its relationship with renal outcomes in patients with AF has not been evaluated. Accordingly, this study assessed whether the E/E’sr ratio is a useful parameter in the prediction of progression to the renal endpoint (a ≥ 25% decline in eGFR) in patients with AF.

## Methods

### Study participants

This observational cohort study prospectively and consecutively included patients with persistent AF referred for echocardiographic examinations to Kaohsiung Municipal Hsiao-Kang Hospital from April 2010 to July 2012. Persistent AF is defined as AF lasting 7 days, which is confirmed using 12-lead electrocardiography, 24-hour Holter electrocardiography, or electrocardiographic recording during echocardiographic examination. Patients with moderate and severe mitral stenosis, moderate and severe aortic stenosis or regurgitation, severe mitral regurgitation, or inadequate echocardiographic visualization were excluded. In addition, patients with fewer than three eGFR measurements during the follow-up period and patients without a follow-up of more than 6 months after enrollment were also excluded to avoid incomplete observation of changes in the renal function. Finally, 149 patients (mean age 69.8 ± 9.9 years, 104 male) were included in this study.

### Ethics statement

The study protocol was approved by the Institutional Review Board of Kaohsiung Medical University Hospital (KMUH-IRB-20130062). Written informed consent was obtained from the patients, and all clinical investigations were conducted according to the principles expressed in the Declaration of Helsinki. The patients also consented to the publication of the clinical details.

### Echocardiographic evaluation

An experienced cardiologist performed echocardiographic examination on participants respiring quietly in the left decubitus position by using Vivid 7 (GE Vingmed Ultrasound AS, Horten, Norway). The cardiologist was blinded to the clinical data, such as history of hypertension, diabetes mellitus, and coronary artery disease. Two-dimensional and anatomic M-mode images were recorded from the standardized views. The Doppler sample volume was placed at the tips of the mitral leaflets to obtain the LV inflow waveforms from the apical four-chamber view. Pulsed Doppler tissue imaging was performed with the sample volume placed at the lateral and septal corners of the mitral annulus to obtain waveforms from the apical four-chamber view. Early diastolic mitral annular velocity (E’) was averaged from measurements at septal and lateral annuli. The wall filter settings were adjusted to exclude high-frequency signals and the gain was minimized. The LV ejection fraction (LVEF) was measured using the modified Simpson’s method, the LV mass was calculated using the modified Devereux method [14], the LV mass index (LVMI) was calculated by dividing the LV mass by the body surface area, the left atrial volume was measured using the biplane area-length method [15], and the left atrial volume index (LAVI) was calculated by dividing the left atrial volume by the body surface area.

LV apical four-chamber, two-chamber, and long-axis views were acquired using a high frame rate (50–90 frames/s). The endocardial border was manually defined using a point-and-click technique. An epicardial surface tracing was automatically generated by the system, creating a region of interest, which was manually adjusted to cover the full thickness of the myocardium in the systolic frame. The ventricular chamber was divided into six segments, and six segmental strain and strain rate curves were analyzed in each apical view. Peak segmental longitudinal systolic strain and early diastolic strain rate measurements were determined from these curves. E’sr was assessed in the 18 LV segments from the three standard apical views. The values in the 18 LV segments were averaged to obtain the mean value for later analysis. The minimum number of LV segments for acceptable E’sr measurements was 15. We used cine loops to determine the beat to be calculated. The raw ultrasonic data, including 15 consecutive beats from the three standard apical views, were recorded and analyzed offline by using EchoPAC version 08 (GE Vingmed Ultrasound AS).

The index beat was used to measure LV dimensions, the LVEF, LAVI, LVMI, and E’sr [9, 16, 17]. Because their measurements were easy and rapid, E, E-wave deceleration time (EDT), and E’ were obtained from five beats [18] and then averaged for later analysis. If the cardiac cycle length was too short to complete the diastolic process, this beat was skipped. Thus, the selection of E, EDT, and E’ was not always consecutive. In addition, the heart rate was determined from five consecutive beats.

### Collection of demographic, medical, and laboratory data

Demographic and medical data including age, sex, and comorbid conditions were obtained from medical records and interviews with patients. The body mass index (BMI) was calculated as the ratio of weight in kilograms divided by the square of height in meters. Laboratory data were measured from fasting blood samples using an autoanalyzer (Roche Diagnostics GmbH, D-68298 Mannheim COBAS Integra 400). Serum creatinine was measured using the compensated Jaffé (kinetic alkaline picrate) method in a Roche/Integra 400 Analyzer (Roche Diagnostics, Mannheim, Germany) using a calibrator traceable to isotope-dilution mass spectrometry [19]. The value of eGFR was calculated using the four-variable equation in the Modification of Diet in Renal Disease (MDRD) study [20]. Dipsticks (Hema-Combistix, Bayer Diagnostics) were used to examine proteinuria. A test result of 1+ or more was defined as positive. Blood and urine samples were obtained within 1 month of enrollment. In addition, information regarding antihypertensive and other medications used by the patients during the study period, including angiotensin converting enzyme inhibitors (ACEI), angiotensin II receptor blockers (ARB), β-blockers, calcium channel blockers, diuretics, and antiplatelet and anticoagulant drugs, was obtained from the medical records.

### Definition of the renal endpoint

The renal endpoint was defined as a ≥ 25% decline in eGFR since the enrollment of the patients [21]. For patients who were reaching the renal endpoint, the renal function data were censored. The patients who did not reach renal endpoints were followed up until the last serum creatinine measurement.

### Statistical analysis

Statistical analysis was performed using SPSS version 17.0 (SPSS Inc., Chicago, IL, USA) for Windows. Data are expressed as percentages, mean ± standard deviation, or median (25^th^-75^th^ percentile) for triglyceride and several serum creatinine measurements. The differences between groups were checked using a chi-squared test for categorical variables and independent t-test for continuous variables. The study participants were stratified into three groups according to tertiles of the E/E’sr ratio. Tertile 1 was considered reference category. Multiple comparisons among the study groups were performed using one-way analysis of variance (ANOVA), followed by a post hoc test adjusted with a Bonferroni correction. Cox proportional hazards analyses were performed to investigate the associations of E/E’sr ratio tertiles and the E/E’sr ratio (continuous data) with progression to the renal endpoint. The adjusted covariates included age, sex, diabetes mellitus, hypertension, coronary artery disease, systolic and diastolic blood pressures, the BMI, fasting glucose, log triglyceride, total cholesterol, hemoglobin, baseline eGFR, uric acid, proteinuria, antihypertensive drug use, antiplatelet agents, anticoagulants, the LAVI, LVMI, LVEF, and EDT. Survival curve for the renal endpoint was derived using Kaplan–Meier analysis. A direct comparison between the E/E’ and E/E’sr ratios was performed in both univariate and multivariate models. Incremental model performance was assessed using a change in the χ^2^ value. A difference was considered significant at *P* < 0.05.

## Results

One hundred and forty-nine patients were included in this study. The mean age of the patients was 69.8 ± 9.9 years; 104 patients were male and 45 were female. During the follow-up period, the average number of serum creatinine measurements was nine (25^th^-75^th^ percentile: 6–15). The E/E’s ratio was 63.0 ± 21.8 cm. The comparison of baseline characteristics and echocardiographic parameters between patients reaching and not reaching the renal endpoint is shown in Table 1. Sixty-three patients (42.3%) reached the renal endpoint. Compared with patients without renal endpoint, patients with a renal endpoint were observed to be older and had higher uric acid, LVMI, EDT, and E/E’sr ratio, and lower LVEF and E’sr.

**Table 1:** Comparison of baseline characteristics between patients with and without renal end point of ≧25% decline in eGFR.

Characteristics	All patients (n = 149)	Patients without renal end point (n = 86)	Patients with renal end point (n = 63)
Age (year)	69.8 ± 9.9	68.0 ± 10.0	72.3 ± 9.4*
Male gender (%)	69.8	67.4	73.0
Diabetes mellitus (%)	28.9	24.4	34.9
Hypertension (%)	67.8	67.4	68.3
Coronary artery disease (%)	10.7	9.3	12.7
Systolic BP (mmHg)	131.0 ± 18.4	131.9 ± 18.3	129.4 ± 18.6
Diastolic BP (mmHg)	76.6 ± 12.2	77.5 ± 12.6	75.2 ± 11.7
Body mass index (kg/m^2^)	26.7 ± 4.2	27.0 ± 4.0	26.2 ± 4.4
Laboratory parameters			
Fasting glucose (mg/dL)	118.8 ± 38.3	119.9 ± 39.4	117.3 ± 37.0
Triglyceride (mg/dL)	101.5 (72.75–147)	97.5 (72–137.25)	115.5 (73.25–172.5)
Total cholesterol (mg/dL)	172.0 ± 34.4	175.8 ± 36.0	166.3 ± 31.4
Hemoglobin (g/dL)	13.7 ± 2.1	13.9 ± 2.1	13.4 ± 2.0
Baseline eGFR (mL/min/1.73 m^2^)	54.4 ± 15.5	56.6 ± 14.3	53.2 ± 17.1
Uric acid (mg/dL)	7.5 ± 2.2	7.1 ± 1.9	8.0 ± 2.5*
Proteinuria (%)	32.6	28.4	38.1
Medications			
ACEI or ARB use (%)	63.1	59.3	68.3
β-blocker use (%)	50.3	54.7	44.4
Calcium channel blocker use (%)	38.3	36.0	41.3
Diuretics use (%)	45.6	40.7	52.4
Antiplatelet agents use (%)	63.1	61.6	65.1
Anticoagulants use (%)	30.2	33.7	25.4
Echocardiographic data			
LAVI (ml/m^2^)	47.6 ± 18.8	46.7 ± 16.2	48.8 ± 22.1
LVMI (g/m^2^)	137.8 ± 41.5	126.3 ± 32.5	153.5 ± 47.3**
LVEF (%)	53.7 ± 14.3	56.0 ± 13.0	50.6 ± 15.4*
E (cm/sec)	95.4 ± 21.6	94.7 ± 20.1	96.3 ± 23.6
EDT (msec)	145.3 ± 42.4	138.7 ± 28.2	154.2 ± 55.1*
E’ (cm/sec)	9.0 ± 2.3	9.2 ± 2.1	8.7 ± 2.4	±
E/E’ ratio	11.2 ± 3.9	10.7 ± 3.2	11.9 ± 4.6
E’sr (sec^-1^)	1.6 ± 0.4	1.7 ± 0.4	1.5 ± 0.4*
E/E’sr ratio (cm)	63.0 ± 21.8	58.1 ± 14.4	69.7 ± 27.8*

Abbreviations. BP, blood pressure; eGFR, estimated glomerular filtration rate; ACEI, angiotensin converting enzyme inhibitor; ARB, angiotensin II receptor blocker; LAVI, left atrial volume index; LVMI, left ventricular mass index; LVEF, left ventricular ejection fraction; E, peak early transmitral filling wave velocity; EDT, E-wave deceleration time; E’, early diastolic velocity of lateral mitral annulus; E’sr, global diastolic strain rate.

*P < 0.05

**P < 0.001 compared with patients without renal end point.

The study participants were stratified into three groups according to tertiles of the E/E’sr ratio (< 51.9, ≥ 51.9 to < 66.2, and ≥ 66.2 cm). A comparison of the clinical characteristics and echocardiographic parameters among the study groups is shown in Table 2. The three groups had 49, 50, and 50 patients. Significant differences in LVMI, LVEF, E, E’, E/E’ ratio, E’sr, and E/E’sr ratio were observed among patients in the different tertiles.

**Table 2:** Comparison of clinical and echocardiographic characteristics among patients according to teriles of E/E’sr ratio.

Characteristics	Tertile 1 (< 51.9) (n = 49)	Tertile 2 (51.9–66.2) (n = 50)	Tertile 3 (≧66.2) (n = 50)	*P*
Age (year)	70.0 ± 10.4	68.7 ± 8.2	70.7 ± 11.0	0.575
Male gender (%)	65.3	66.0	78.0	0.300
Diabetes mellitus (%)	26.5	26.0	34.0	0.615
Hypertension (%)	59.2	70.0	74.0	0.265
Coronary artery disease (%)	8.2	6.0	18.0	0.119
Systolic BP (mmHg)	130.4 ± 19.3	130.4 ± 19.2	132.1 ± 16.9	0.888
Diastolic BP (mmHg)	76.7 ± 12.9	76.1 ± 11.6	76.9 ± 12.5	0.955
Body mass index (kg/m^2^)	26.4 ± 3.2	26.9 ± 4.4	26.7 ± 4.9	0.858
Laboratory parameters				
Fasting glucose (mg/dL)	114.2 ± 32.9	121.2 ± 39.1	121.0 ± 42.8	0.675
Triglyceride (mg/dL)	104.5 (73.25–147)	103 (76.25–153.5)	94.5 (64.75–137.75)	0.766
Total cholesterol (mg/dL)	175.0 ± 42.4	176.4 ± 29.2	164.5 ± 29.9	0.249
Hemoglobin (g/dL)	13.9 ± 1.8	13.8 ± 2.2	13.3 ± 2.1	0.359
Baseline eGFR (mL/min/1.73 m^2^)	54.6 ± 15.9	54.4 ± 13.9	60.2 ± 16.1	0.101
Uric acid (mg/dL)	7.1 ± 2.1	7.2 ± 1.9	8.0 ± 2.4	0.097
Proteinuria (%)	19.6	39.6	38.0	0.071
Medications				
ACEI or ARB use (%)	73.5	54.0	62.0	0.131
β-blocker use (%)	53.1	52.0	46.0	0.749
Calcium channel blocker use (%)	40.8	34.0	40.0	0.747
Diuretics use (%)	49.0	34.0	54.0	0.113
Antiplatelet agents use (%)	67.3	56.0	66.0	0.440
Anticoagulants use (%)	20.4	36.0	34.0	0.186
Echocardiographic data				
LAVI (ml/m^2^)	47.6 ± 21.4	45.8 ± 11.5	49.2 ± 21.9	0.680
LVMI (g/m^2^)	127.4 ± 38.4	134.0 ± 34.7	151.3 ± 47.2*	0.014
LVEF (%)	60.5 ± 9.5	55.2 ± 12.8	45.5 ± 15.8*^†^	< 0.001
E (cm/sec)	84.8 ± 15.7	95.9 ± 17.0*	105.2 ± 25.9*	< 0.001
EDT (msec)	137.3 ± 28.9	143.9 ± 28.1	154.5 ± 60.3	0.128
E’ (cm/sec)	9.9 ± 2.2	9.3 ± 2.1	7.8 ± 2.0*^†^	< 0.001
E/E’ ratio	8.9 ± 2.4	10.6 ± 2.2*	14.1 ± 4.7*^†^	< 0.001
E’sr (sec^-1^)	1.9 ± 0.4	1.6 ± 0.3*	1.3 ± 0.3*^†^	< 0.001
E/E’sr ratio (cm)	44.4 ± 4.3	58.5 ± 4.4*	85.5 ± 22.8*^†^	< 0.001

Abbreviations. BP, blood pressure; eGFR, estimated glomerular filtration rate; ACEI, angiotensin converting enzyme inhibitor; ARB, angiotensin II receptor blocker; LAVI, left atrial volume index; LVMI, left ventricular mass index; LVEF, left ventricular ejection fraction; E, peak early transmitral filling wave velocity; EDT, E-wave deceleration time; E’, early diastolic velocity of lateral mitral annulus; E’sr, global diastolic strain rate.

*P <0.05 compared with Tertile 1.

^†^*P* < 0.05 compared with Tertile 2.

### Risk of progression to the renal endpoint

**Fig 1:**
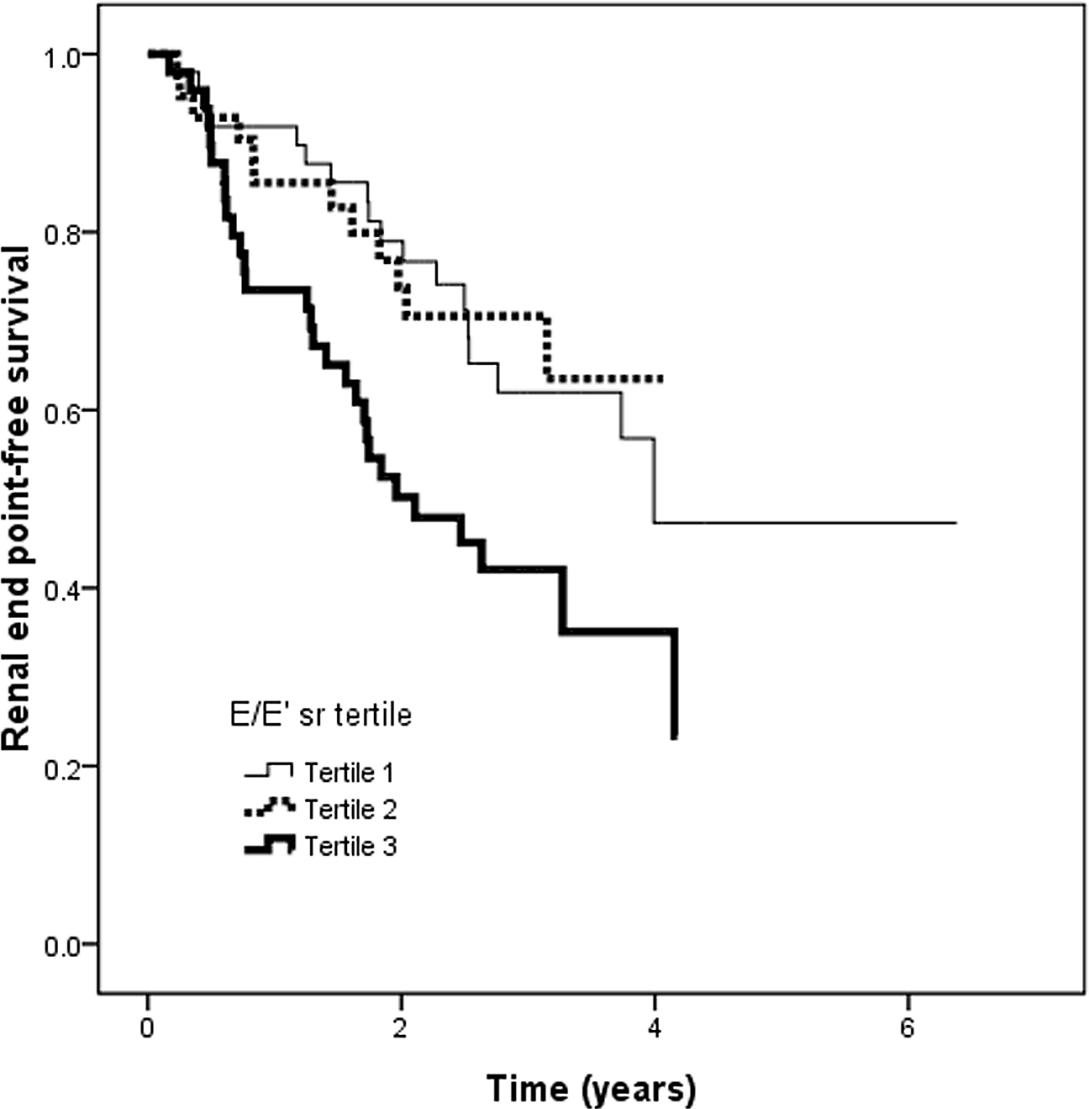
Kaplan-Meier analysis of renal end point-free survival according to tertiles of E/E’sr ratio (log-rank *p* = 0.008). Patients with tertile 3 of E/E’sr ratio had a worse renal end point-free survival than those with tertile 1 of E/E’sr ratio.

The mean follow-up period was 2.3 ± 1.2 years. During the follow-up period, 63 patients (42.3%) reached the renal endpoint. Table 3 lists the hazard ratios (HRs) of the E/E’sr ratio tertiles and E/E’sr ratio for the renal endpoint with and without adjustment of the demographic, clinical, and biochemical factors. Patients with tertile 3 of the E/E’sr ratio (versus tertile 1) were associated with an increased renal endpoint in an unadjusted model (HR, 2.161; 95% confidence interval [CI], 1.197 to 3.900; *p* = 0.011) and adjusted model (HR, 6.148; 95% CI, 1.990 to 18.993; *p* = 0.002). Fig 1 illustrates the Kaplan–Meier curves for renal endpoint-free survival (log-rank *p* = 0.008) in all patients subdivided according to tertiles of the E/E’sr ratio. A similar pattern of association was observed using the E/E’sr ratio as a continuous variable. An increased E/E’sr ratio was associated with an increased renal endpoint in an unadjusted model (HR, 1.190; 95% CI, 1.087 to 1.302; *p* < 0.001) and an adjusted model (HR, 1.230; 95% CI, 1.088 to 1.391; *p* = 0.001).

**Table 3:** Relation of E/E’sr tertiles and E/E’sr (continuous data) to progression to renal end point (≧25% decline in eGFR) using Cox proportional hazards model.

Parameter	Unadjusted		Adjusted (forward)	
	HR (95% CI)	*P*	HR (95% CI)	*P*
E/E’sr				
Tertile 1	1		1	
Tertile 2	0.819 (0.412–1.628)	0.570	1.023 (0.278–3.761)	0.973
Tertile 3	2.161 (1.197–3.900)	0.011	6.148 (1.990–18.993)	0.002
E/E’sr (per 10 cm)	1.190 (1.087–1.302)	< 0.001	1.230 (1.088–1.391)	0.001

Values express as hazard ratios (HR) and 95% confidence interval (CI).

Adjusted for age, sex, diabetes mellitus, hypertension, coronary artery disease, systolic and diastolic blood pressures, body mass index, fasting glucose, log triglyceride, total cholesterol, hemoglobin, baseline eGFR, uric acid, proteinuria, anti-hypertensive drugs use, antiplatelet agents, anticoagulants, LAVI, LVMI, LVEF and EDT.

The use of ACEIs or ARBs was not significantly associated with an increased renal endpoint in univariate analysis (HR, 1.338; 95% CI, 0.785 to 2.280; *p* = 0.284).

### Comparison of the E/E’ and E/E’sr ratios in progression to the renal endpoint

In the univariate analysis, the E/E’sr ratio was significantly related to the renal endpoint and outperformed the E/E’ ratio of the model in a direct comparison (χ^2^ = 14.24 *vs*. 6.113, *p* = 0.004). In the multivariate analysis, an increased E/E’sr ratio was independently associated with an increased renal endpoint. In a direct comparison, the multivariate model without E/E’ and E/ E’sr ratios (global χ^2^ = 46.91) was not significantly improved by adding the E/E’ ratio (globalχ^2^ = 47.29, *p* = 0.538), whereas adding the E/E’sr ratio resulted in significant improvement (globalχ^2^ = 51.15, *p* = 0.039).

### Incremental value of the E/E’sr ratio in relation to the renal endpoint

**Fig 2:**
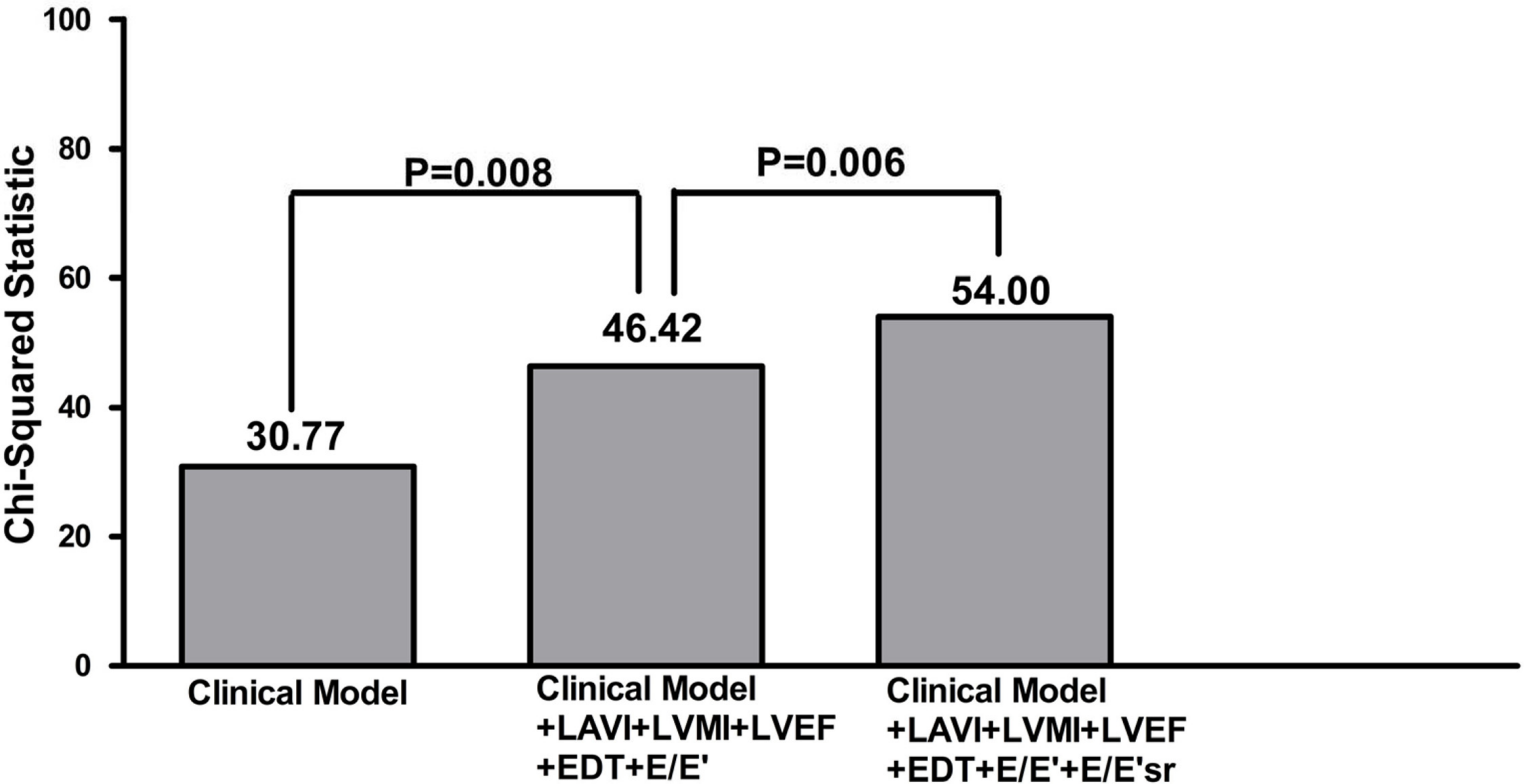
Addition of the ratio of E to E’sr to a Cox model including clinical variables, LAVI, LVMI, LVEF, EDT and E/E’ resulted in a significant improvement in the prediction of progression to renal end point of ≧25% decline in eGFR (*p* = 0.006).

The incremental value of the E/E’sr ratio in the outcome prediction is shown in Fig 2. The clinical model includes age, sex, diabetes mellitus, hypertension, coronary artery disease, systolic and diastolic blood pressures, the BMI, fasting glucose, log triglyceride, total cholesterol, hemoglobin, baseline eGFR, uric acid, proteinuria, antihypertensive drug use, antiplatelet agents, and anticoagulants (χ^2^ = 30.77). Adding the LAVI, LVMI, LVEF, EDT, and E/E’ ratio to the clinical model offered an additional benefit in the prediction of progression to the renal endpoint (*p* = 0.008). In addition, adding the E/E’sr ratio to the model consisting of the clinical model, LAVI, LVMI, LVEF, EDT, and E/E’ ratio resulted in further significant improvement in the prediction of the renal endpoint (*p* = 0.006).

## Discussion

In this study, we evaluated the association of the E/E’sr ratio with the progression to the renal endpoint in patients with AF. We observed that the increased E/E’sr ratio was independently associated with an increase in the renal endpoint in patients with AF. In a direct comparison, the E/E’sr ratio outperformed the E/E’ ratio in predicting progression to the renal endpoint in both univariate and multivariate models. Furthermore, the E/E’sr ratio could add a significant incremental prognostic value beyond the conventional clinical and echocardiographic parameters.

The first major finding of our study was the identification of the E/E’sr ratio as a risk factor for progression to the renal endpoint in patients with AF. Recent studies have demonstrated that CKD patients with an increased LV filling pressure indicated by an increased LAVI, left atrial diameter, and E/E’ ratio from tissue Doppler echocardiography might have adverse renal outcomes [6, 22, 23]. In a recent study, we evaluated the association between the E/E’ and renal dysfunction progression, and observed that patients with a rapid renal function decline had a higher E/E’ [8]. Furthermore, a high E/E’ was independently associated with increased risk of commencement of dialysis in CKD patients [6]. In addition, our study revealed that increased E/E’sr was associated with an increase in the renal endpoint in patients with AF. Kusunose et al. [9] have demonstrated that the E’sr measured using the index beat in patients with AF is an accurate estimate of the E’sr measured from averaging multiple cardiac cycles and is significantly correlated with a time constant of isovolumic left ventricular pressure decay. In addition, they observed that E/E’sr was highly associated with LV filling pressure in patients with AF. This implied that AF patients with a high E/E’sr ratio might have a high volume status, thereby increasing renal efferent pressure and decreasing renal blood flow and finally causing a progressive renal function decline [24]. The higher preload status might contribute to a more rapid renal progression. Hence, an assessment of the E/E’sr ratio in patients with AF may facilitate identifying the high-risk group with adverse renal outcomes.

The second major finding of our study was that the E/E’sr ratio was a useful predictor and was stronger than the E/E’ ratio in predicting progression to the renal endpoint in patients with AF. Recently, we reported that the E/E’sr ratio is a useful parameter and is stronger than the E/E’ ratio in predicting adverse cardiac events in patients with AF [13]. E’ was developed to assess regional diastolic function, and its value for estimating a global diastolic function with regional functional abnormalities is limited [25]. In addition, the inherent limitations of Doppler-based methods, such as angle dependency with the potential for significant errors with angulations > 20°, may also preclude E’ from reflecting a truly early diastolic function. By contrast, E’sr derived from two-dimensional speckle-tracking echocardiography was recently introduced as a novel parameter to reflect LV relaxation function in patients with AF [9]. This imaging method discriminates between the active and passive myocardial motion and enables the angle-independent quantification of myocardial deformation in two dimensions. Hence, E’sr should be able to reflect the LV diastolic function more globally and accurately than E’ can.

The third major finding of this study was that adding the E/E’sr ratio to the model consisting of clinical parameters, the LAVI, LVEF, LVMI, EDT, and E/E’ ratio provided an additional benefit in the prediction of poor renal prognosis. Our previous data showed that concentric LVH was associated with progression to dialysis, and that an increased LA diameter and a decreased LVEF and midwall fractional shortening were associated with a rapid renal function decline [7]. In addition, Furukawa et al. [23] evaluated the LAVI factor in progression to hemodialysis in 140 patients with CKD stage 4–5. They observed that the LAVI was a risk factor for the period before dialysis. In our study, although conventional clinical and echocardiographic systolic and diastolic parameters were known, considering the E/E’sr ratio facilitated improving the renal outcome prediction in patients with AF. The E/E’sr ratio should be measured during echocardiographic examination for additional prognostication in these patients.

There were several limitations to this study. Because their measurement using the averaging method is time consuming, many echocardiographic parameters, including the LV dimensions, LVEF, LAVI, LVMI, and E’sr, were not obtained by averaging multiple cardiac cycles but were measured according to the index beat. However, several studies have proved that using the index beat to measure certain echocardiographic parameters, including LV systolic and diastolic functions and left atrial parameters, in patients with AF is as accurate as the time-consuming method for averaging multiple cardiac cycles [9, 16, 17]. Two-dimensional speckle-tracking echocardiography generated longitudinal, radial, and circumferential deformation measurements and LV twist. In this study, only E’sr was measured and analyzed. The majority of our patients were receiving long-term treatment involving antihypertensive, antiplatelet, and anticoagulant medications. For ethical reasons, we did not withdraw these medications. Hence, we could not exclude the influence of these medications on the present findings. However, we adjusted for the use of these medicines in the multivariate analysis. Because the subjects of this study were already being evaluated for heart disease by echocardiography, it was susceptible to selection bias, making our findings potentially less generalized.

In conclusion, our results demonstrated that the E/E’sr ratio is a useful parameter and is stronger than the E/E’ ratio in predicting adverse renal outcomes in patients with AF, and it may offer an additional prognostic benefit over conventional clinical and echocardiographic parameters.

## Supporting Information

S1 File Relevant data including E/E’ sr and renal outcome. (XLS)
